# History of insulin

**DOI:** 10.3402/jchimp.v2i2.18701

**Published:** 2012-07-16

**Authors:** Celeste C. Quianzon, Issam Cheikh

**Affiliations:** Department of Medicine, Union Memorial Hospital, Baltimore, Maryland, USA

**Keywords:** diabetes medication, history, review

## Abstract

The advancement of diabetes treatment has gone from crude extracts of insulin and accidental discovery of sulfa-like drugs in antibiotics to the development of drugs based on improved understanding of the pathophysiology of diabetes mellitus. This article will review the history of the discovery and development of insulin. A companion focusing on non-insulin diabetes agents will follow in the next issue of JCHIMP.

The earliest description of diabetes appeared in a collection of medical texts in Egypt written around 552 BC, the *Ebers Papyrus* (1, 2) . Diabetes mellitus and its medicinal remedies were described in ancient India and China (1). Aretaeus of Cappadocia, a Greek physician, (129–199 AD) introduced the term “diabetes” from the Greek word “siphon” as he noted that diabetes causes constant flow of urine (2, 3). Before the availability of insulin, the life expectancy of children with diabetes mellitus was short and the prognosis for the adult onset diabetes was very poor.

## Before insulin

During the early part of the 20th century, before insulin became available, physicians Allen and Joslin endorsed fasting and calorie-restricted diets for diabetes (4). This resulted in some improvement of glucosuria and acidosis, decreased coma, and delayed death among children with diabetes. All diabetics were advised to decrease their sugar and dietary starch intake, and those who were obese were advised to lose weight.

## Insulin

The discovery of insulin in 1922 marked a major breakthrough in medicine and therapy in patients with diabetes. Long before the discovery of insulin, it was hypothesized that the pancreas secreted a substance that controlled carbohydrate metabolism (5). For years, attempts at preparing pancreatic extracts to lower blood glucose were unsuccessful due to impurities and toxicities (6). Frederick Banting, an orthopedic surgeon, had the idea of isolating pancreatic islet extracts by ligating the pancreatic duct of dogs, keeping them alive until the acini degenerated, leaving the islets for isolation. He approached John Macleod, professor of physiology and department head at the University of Toronto, for laboratory space. Macleod granted him laboratory space, ten dogs for his experiments, a student research assistant (Charles Best), and provided supervision and guidance. The experiments began on May 17, 1921, and by September they showed that the depancreatized dog developed diabetes and that intravenous injection with their pancreatic extract, which they named *isletin*, lowered the blood glucose. By late 1921, the biochemist J.B. Collip joined the group and helped purify the isletin for human use. The first injection of the pancreatic extract to a 14-year-old boy by Banting and Best on January 11, 1922, caused a sterile abscess, had no effect on ketosis, and resulted in mild blood glucose reduction. Subsequent injections of the purified extract by Collip had promising results that same year. Blood glucose and glucosuria decreased, and ketonuria disappeared. Rosenfeld reported encouraging results in six more patients (6). Several months later, in 1923, Banting, Best, and Macleod were awarded the Nobel Prize.

Eli Lilly began producing insulin from animal pancreas but fell short of the demand, and the potency varied up to 25% per lot (6). The development of an isoelectric precipitation method led to a purer and more potent animal insulin, decreasing the variation between lots to 10% (6).

In 1923, August Krogh, from the University of Copenhagen, met with Banting and Macleod to learn more about insulin because his wife had diabetes mellitus. He received authorization from the University of Toronto to bring insulin to Scandinavia. A non-profit body, Nordisk Insulin Laboratory, began insulin production (6).

Because the insulin preparation required several injections daily, investigators worked to find ways to prolong its duration of action. In the 1930s, H.C. Hagedorn, a chemist in Denmark, prolonged the action of insulin by adding protamine (5). In Toronto, Scott and Fisher prolonged insulin action further by adding zinc (5). These discoveries led to the introduction of longer-acting animal insulins in the market. Protamine zinc insulin lasted 24–36 hours. Isophane neutral protamine Hagedorn lasted 24 hours and could be mixed with regular insulin. The pharmacokinetics and effects of amorphous lente insulin (semilente, lente, and ultralente) depended on the proportion of zinc. In 1978, the first recombinant DNA human insulin was prepared by David Goeddel and his colleagues (of Genentech) by utilizing and combining the insulin A- and B- chains expressed in *Escherichia coli*. Thereafter, Genentech and Lilly signed an agreement to commercialize rDNA insulin. In 1982, the first insulin utilizing rDNA technology, Humulin^®^ R (rapid) and N (NPH, intermediate-acting), were marketed.

Once patients with diabetes started to live longer, chronic complications of diabetes became prevalent. In 1993, the Diabetes Control and Complications Trial showed for the first time without any doubt the linear relation between the degree of glycemic control and complications (8). To reduce the incidence of hypoglycemia, which is the major limiting factor for intensive glycemic control, physiologic insulins that mimic the basal and prandial insulin secretion were sought. Modification of the site of amino acids in the insulin changed the pharmacokinetics and led to faster absorption, earlier peak of action, and shorter duration of action (9). Lispro was the first short-acting insulin analog approved in 1996 (10) followed by aspart in 2000 (11) and glulisine in 2004 (12). Currently, there are two basal insulin analogs in the market, glargine, approved in 2000 (13) and detemir, approved in 2005 (14). Glargine has glycine instead of asparagine at position A21, an extra two arginine molecules at position B30 and a pH of 4.0. It forms microprecipitates at the site of injection resulting in a prolonged absorption with little peak activity (9, 15). Insulin detemir has a 14-carbon fatty acid chain attached to lysine at position B29 which slows its absorption (16).

To have an alternative delivery method for insulin, exubera, the first inhaled insulin, was developed by Sanofi-Aventis and Pfizer and marketed by Pzifer in 2006 (17). The inhaler device was bulky to use. It did not add physiologic benefit over rapid-short acting insulin analogs (18). It was taken off the market after two years when it failed to gain acceptance from patients and providers (17, 19).

## Conclusion

The discovery of pancreatic crude extracts gave hope to patients with diabetes mellitus. The subsequent development of precisely engineered insulin analogs, which are more physiologic, improved diabetes control and reduced or delayed complications. Insulin continues to be the cornerstone of therapy. Newer medications complement and enhance insulin action tailored toward different mechanisms in the pathophysiology of diabetes mellitus.

Meanwhile, in the next issue of JCHIMP, we will review the other agents for diabetes care.
